# Strategies for Diagnosis and Prevention of Venous Thromboembolism during Pregnancy

**DOI:** 10.1155/2011/206858

**Published:** 2011-07-21

**Authors:** Shalini Jain Bagaria, V. B. Bagaria

**Affiliations:** Health Care Research and Innovation Division, Origyn Clinic, Indirapuram, Ghaziabad 201014, India

## Abstract

Pregnancy and the postpartum period have an increased incidence of venous thromboembolism (VTE). The condition is unique during this period for several reasons. Primarily, because there is complexity in diagnosing this condition in view of altered physiology and preexisting edema in pregnancy and also because there are restrictions on the use of certain drugs and a need for vigilant monitoring of anticoagulant activities of drugs during the period. The problem is compounded and assumes the highest order of significance since two lives are involved and all the investigations and management done should also take into account the potential adverse effects on the foetus. In order to prevent the development of VTE during pregnancy, sound clinical evaluation for risk factors, risk stratification, and optimal use of resource both mechanical and pharmacological is necessary. This paper details strategies in preventing development of deep vein thrombosis and treatment of VTEs.

## 1. Introduction

Venous thromboembolism is used to denote two disease conditions: deep vein thrombosis (DVT) and pulmonary embolism (PE). The two conditions are interrelated in a manner that approximately one third of all cases of isolated PE will have DVT on diagnostic evaluation and similarly approximately half the cases of DVT of proximal veins of leg will have clinical silent or apparent PE [1–3]. VTE assumes a special significance during pregnancy due to its increased incidence, difficulty in diagnosis, and complexities in management. 

The incidence of VTE in pregnancy is approximately five times the incidence in nonpregnant patients and is reported to be 0.7 to 1.2 per 10000 pregnancies [4, 5]. This risk increases to approximately 20 times in the postpartum period. DVT constitutes almost 80% of cases of VTE, and the rest are constituted by PE. It also accounts for 10% maternal deaths in the western world or roughly 1.1 deaths per 100 000 deliveries [6].

Virchow in 1856 suggested that development of PE could be attributed to stasis, endothelial injury, and hypercoagulable state [7]. Over the years, various research investigations have upheld his observation. Pregnancy is classified as a hypercoagulable state as the fibrin generation is increased, fibrinolytic activity is decreased, levels of coagulation factors II, VII, VIII, and X are all increased, free protein S levels are decreased, and acquired resistance to activated protein C is commonly seen [8, 9]. During normal uncomplicated pregnancy, there is also an evidence of substantial haemostatic activation as indicated by increased markers of coagulation activation, such as prothrombin fragment F1+2 and D-dimer [9]. Also, there is hormonally induced decreased venous capacitance and reduced venous outflow due to mechanical obstruction from uterus [10, 11]. There is 50% decrease in the venous outflow during 26–30 weeks of gestation which continues through 6 weeks postgestation (Table 1). 

Additional risk factors include the presence of inherited thrombophilias and the antiphospholipid syndrome, previous history of thrombosis, black race, heart disease, sickle cell disease, diabetes, lupus, smoking, multiple pregnancy, age greater than 35 years, obesity, and caesarean delivery (especially emergency caesarean section during labor) [12–15] (Table 2).

## 2. Strategies for Diagnosing VTE

### 2.1. Risk Stratification

It is vital that a high degree of suspicion and clinical vigilance be maintained during the pregnancy and postpartum period. It is worth noting that typical signs like leg swelling, tachycardia, tachypnea, and dyspnoea that raise the suspicion of VTE in the nonpregnant are often already present as a response to physiological changes occurring during the pregnancy [16, 17]. All cases of suspicion of VTE should be investigated expeditiously with following available modalities.

### 2.2. Duplex Ultrasonography

The preferred modality for investigation of DVT is duplex ultrasonography which has a sensitivity of approximately 97% and specificity of approximately 94% in general population for proximal venous thromboembolism [18, 19]. It is usually done with a 5 Hz probe and incorporates two elements: gray-scale ultrasound and colour Doppler study. Gray-scale ultrasound is used to visualize the structure or the architecture of the body part. Colour Doppler is used to visualize the flow in the vessel. Normal venous flow produces a low pitched sound that is absent in case of venous occlusion. The ultrasound examination not only helps to determine the anatomy and patency but also can be used to evaluate the augmentation (increased flow with calf compression) and compression (elimination of the residual lumen by firm pressure of the hand-held transducer probe).

Limitations of duplex ultrasound: duplex ultrasound is not very effective in diagnosing asymptomatic and calf vein DVT with only a reported sensitivity of 36% of calf vein VTE [21]. Similarly, for more proximal thrombus like those involving the iliac veins, it may be necessary to perform an MRI direct thrombus imaging as it does not involve radiation and harmful effects on the foetus [22].

### 2.3. D-Dimer

D-dimer is another test which when used with other investigations may help in reaching conclusive diagnosis although the D-dimer level is normally elevated during the pregnancy [23, 24]. This hampers the use of normal reference values [25] to rule out VTE or to monitor antithrombotic treatment when used alone. However, when used in conjunction with other modalities, it can help rule out the condition when in doubt. Nishii et al. in a large prospective study showed that the test had positive predictive value of 7.4% and negative predictive value of 95.5% for ultrasonographically positive women when D-dimer was set at 3.2 microg/mL [26].  Table 3 below gives an indicative range of D-dimer during pregnancy.

### 2.4. Diagnosing Pulmonary Embolism

In case of suspicion of pulmonary embolism (PE), additional investigations need to be performed. An X-ray of chest helps to exclude other causes that lead to dyspnoea and tachycardia. Other investigations of choice are ventilation-perfusion scan and CT pulmonary angiography [27, 28]. Both these tests though routinely used for diagnosing PE in normal population are used with a caution in this group owing to the fact that their use involves radiation exposure to both mother and foetus and have been reported to be associated with increased incidence of childhood cancers and maternal breast cancers.

### 2.5. Ventilation-Perfusion Scan

Ventilation-perfusion scan used to be the more frequently employed modality of investigation in these cases [29]. In pregnant women, the radiation dose can be minimized by using a half-dose perfusion scan and only proceeding to ventilation imaging once an abnormal defect is obtained during the perfusion scan. This test if negative can help confidently exclude the presence of PE; however, the test can indicate an indeterminate results in large number of patients in this group with up to 20% who have been shown to have high probability for the condition not actually having PE.

### 2.6. CT Pulmonary Angiography (CTPA)

CTPA has gradually taken over as the investigation of choice for investigating the cases with suspicion of PE. This is due to the fact that it has high sensitivity (around 94%), high specificity (around 100%), and an excellent negative predictive value of 99% [30, 31]. Unlike V/Q scan, in CTPA, the thrombus can be directly visualised and other alternative cause for the symptoms may also be detected. Another advantage of CTPA over the V/Q scan is the fact that the radiation exposure in CTPA is almost half that of those received during V/q scan. In a typical CTPA, the radiation exposure to foetus is around 3.3 uGy to 130 uGy depending on the trimester the baby is in. 

The risk increases with each passing week as the surface area of the fetus increases leading to an enhanced absorption [32]. Although the fact remains that any radiation exposure to fetus does carry a risk, it is important that, in an indicated case, the investigations are performed expeditiously because the risk of fetal death is high in an untreated case of PE. Apart from the radiation risk to fetus, the risk of radiation exposure to female breast must also be considered. The female breast is extremely radiosensitive, and it has been shown that a sufficiently large radiation dose can cause breast cancer [33, 34]. However, as mentioned above, it should not preclude its use in situations where there is suspicion of PE as the risks involved in terms of radiation exposure are extremely small compared to benefits of the early diagnosis and prompt management of PE.

## 3. Prevention Strategies

### 3.1. Mechanical Prophylaxis

These measures increase the venous blood flow in the lower limbs and prevent venous stasis, an important component of Virchow's triad [35]. Mechanical prophylaxis includes measures such as physiotherapy and exercises, use of graduated compression stockings, foot pumps, and intermittent pneumatic compression devices.

### 3.2. Physiotherapy and Exercises

All patients should have a plan for active and passive lower extremity activity unless contraindicated including flexion and extension of the ankle, knees, and hips. Involve physical therapy as appropriate. Provide written instructions, with pictures as well as a demonstration. Early and aggressive ambulation for all patients if not contraindicated is an important measure to prevent development of VTE.

### 3.3. Graduated Compression Stockings (GCSs)

Encourage patients to wear GCSs at all times except when they are removed for skin care or bathing. Proper size should be instructed, and it should ideally be thigh high to have effect on proximal veins. Nursing staff should ensure that stockings are not leading to a garter effect at the thigh or calf.

### 3.4. Intermittent Pneumatic Compression (IPC)

These should be used in inactive patients requiring passive movements. These devices also exert fibrinolytic effect by stimulating endogenous fibrinolytic mechanism, reducing plasminogen activator inhibitor activity, and increasing the levels of tissue plasminogen activator [36].

### 3.5. IVC Filters

Inferior vena cava filter placement may be indicated in high-risk cases where the use of thromboprophylaxis is contraindicated due to certain coexisting morbidities.

### 3.6. Thromboprophylaxis

Ideally, the evaluation of whether a patient requires thromboprophylaxis should be done before conception or at least early in the pregnancy. Despite a long list of risk factors for development of VTE, most women do not require anticoagulation during pregnancy (Table 4). 

The risk of complications from anticoagulation, such as bleeding, can be as high as 2%. Hence, the use of anticoagulants is limited to cases where the benefits of its use are greater than potential adverse effect (Table 5) [37–44]. In general, pregnant women who have had previous episodes of VTE, those having thrombophilia especially those with antithrombin deficiency, antiphospholipid syndrome, compound heterozygosity for prothrombin G20210A variant or factor V Leiden are candidates for thromboprophylaxis.

### 3.7. Heparin for Thromboprophylaxis

Heparin both unfractionated and low molecular weight heparin are agents of choice for thromboprophylaxis during pregnancy. Neither of them cross placenta making it safe for use in pregnancy. Heparin both UFH and LMWH act by binding to antithrombin to catalyse the molecule binding to and altering the activity of serine protease procoagulants thus interrupting the coagulation pathway [41–44]. UFH enhances the activity of antithrombin for factor Xa and thrombin, whereas the predominant effect of LMWH is via antithrombin-mediated antifactor Xa activity. The dosage and monitoring strategy for UFH is mentioned in Table 6. 

Although there are few direct studies comparing the use of UFH to LMWH during pregnancy, it is widely agreed that the use of LMWH is associated with lower incidence of complications [45, 46]. UFH has complex pharmacokinetics that potentially leads to a somewhat unpredictable anticoagulant response. Also, the bioavailability of the UFH after subcutaneous (SC) injection is reduced compared with intravenous infusion. LMWH, in contrast, is less likely to bind nonspecifically to various circulating protein or cell surfaces and so has improved pharmacokinetics and bioavailability when given SC [47–49]. Other potential advantages include ease of administration, less bleeding, less incidences of heparin-induced thrombocytopenia (HIT), and a predictable response although the therapy is relatively more expensive. Various types of LMWH and their dosage are mentioned in Table 7. One important aspect that should be taken care of during the last trimester of pregnancy is to shift the patients on LMWH to UFH at 36-37 weeks. This is important to prevent development of epidural hematoma, in case an epidural analgesia is planned. In cases of planned caesarean sections, LMWH can be continued even up to 6–12 hrs before surgery. UFH on the other hand has short half life and can be stopped four hours before the epidural insertion with minimal risks and hence is favored over LMWH by many obstetricians towards the term. 

Another newer oral anticoagulant that is being increasingly used is oral anti-Xa inhibitor Rivaroxaban (Trade name: Xarleto). However, there are no adequate data of its use in pregnant women. In fact, studies in animals have shown reproductive toxicity secondary to trans-placental transmission. Due to this potential risk, its use is contraindicated in the pregnancy.

### 3.8. Treatment of VTE Using Heparins in Pregnancy

For managing VTE during pregnancy, two alternative approaches are employed: (1) IV UFH followed by at least 3 months of SC LMWH or adjusted-dose SC UFH or (2) adjusted-dose SC UFH or LMWH can be used both for initial and long-term treatment. With UFH, doses should be adjusted to prolong a midinterval aPTT into the therapeutic range (adjusted-dose SC heparin). As mentioned above, LMWH is the preferred agent amongst the two because of various reasons including better safety profile, ease of administration, and easier monitoring. Since the half life of LMWH is decreased in pregnancy, twice-daily regimens are probably preferable to once-daily dosing and as the pregnancy progresses (and most women gain weight), the potential volume of distribution for LMWH changes requiring change in the dose and pattern of administration. Apart from the usual weight-based regime, some clinicians prefer to perform regular antifactor Xa levels 3 to 4 h after the morning dose and adjust the dose of LMWH to achieve an anti-Xa level of approximately 0.5 to 1.2 U/mL [50, 51].

### 3.9. Use of Warfarin in Pregnancy

Since warfarin crosses placenta, its use is associated with fetal hazards and thus is not recommended during pregnancy. It is associated with high risk of miscarriages with reports indicating rates as high as 56% if taken during first trimester. It has also shown to have 30% risk of congenital anomalies. Warfarin embryopathy is characterized by midface hypoplasia, stippled chondral calcification, scoliosis, short proximal limbs, and short phalanges; it affects 5% of fetuses that are exposed to the drug between 6 and 9 weeks of gestation [52]. The use of warfarin in the second trimester and early in the third trimester is associated with fetal intracranial haemorrhage and schizencephaly [53, 54]. Long-term squeal includes risk of adverse neurological outcome and up to 4% lower IQ. 

There are few conditions in which warfarin may be used preferentially over heparins by certain clinicians. Certain reports have shown that the LMWH may not be as effective as warfarin in protecting mothers from thrombosis of prosthetic valves [55]. Viteale et al. [56] recommend that patient with prosthetic valves whose warfarin intake is 5 mg with an international normalized ratio (INR) within therapeutic range may continue to take warfarin during the entire pregnancy under strict medical surveillance and consider a programmed caesarean section at the 38th week of gestation while briefly interrupting warfarin therapy. On the other hand, those patients whose warfarin doses are >5 mg should be made fully aware of a likely much higher risk of fetal complications during pregnancy. If they decide to carry on pregnancy with warfarin and have a bileaflet or aortic valve prosthesis, the INR range may be lowered to 2.0–2.5 with the aim of bringing the warfarin intake down to 5 mg while still reaching a satisfactory antithrombotic effect. In those women who choose not to take warfarin and are at higher thrombotic risk (mitral prostheses, atrial fibrillation, first-generation valves, and previous thromboembolism), in-hospital heparin treatment, at least between weeks 6 and 12 and 2 weeks before delivery, seems justified. Warfarin for thromboprophylaxis during the postpartum period may be considered where the adverse effect to fetus is not a concern. Warfarin is not secreted in breast milk and thus can be safely given during this phase.

### 3.10. Management in Case of Suspicion of VTE

In cases where the VTE is suspected, the management depends on the degree of clinical suspicion and the stage of pregnancy. The management will also change if certain anticoagulants are contraindicated and whether DVT, PE, or both are suspected. In cases where there is a strong suspicion of an acute episode of pulmonary embolism, it is advisable to start anticoagulant therapy even before the diagnostic evaluation. It may be discontinued if the diagnostic evaluation refutes the diagnosis. In cases of low to moderate degree of suspicion of PE, it is better to evaluate the patient clinically and diagnostically before the anticoagulant therapy is instituted. 

In cases where there is a strong suspicion of PE and anticoagulant therapy is contraindicated, a diagnostic evaluation is warranted without any waste of time. The basic management include supportive management and timely delivery of the fetus. Once the PE is confirmed, other measures especially like IVC filter may be needed. In case of concern for isolated DVT without PE, it is better to diagnostically evaluate the patient before anticoagulant therapy is instituted.

## 4. Conclusion

For patients with risk factors, general thromboprophylaxis, such as lower extremity exercise on a bed; GCS, Intermittent Pneumatic compression (IPC), and adequate hydration postpartum, are recommended. Early ambulation should be encouraged even after a normal delivery in low-risk patients. In addition, pharmacologic thromboprophylaxis with LMWH or warfarin should be considered in patients with risk factors other than caesarean section. For high-risk pregnancies with documented thrombophilia such as positive antiphospholipid antibody or previous VTE, pharmacologic thromboprophylaxis with LMWH is recommended. Warfarin is contraindicated during pregnancy (category X). However, warfarin can replace LMWH after delivery and be used for 6 weeks to 3 months for continued postpartum thromboprophylaxis.

## Figures and Tables

**Table 1:** Causes for increased incidence of VTE during pregnancy.

(1) Hypercoagulation state in pregnancy
(a) Increase in the levels of procoagulants
(i) Factor II, factor VII, factor X, and fibrin
(2) Anticoagulants decrease
(a) Acquired protein C resistance
(b) Decreased levels of protein S
(3) Increased venous stasis
(a) Decreased venous capacitance under hormonal influence
(b) Increased intravascular volume distends veins
(c) Inferior vena cava obstructed secondary to pressure from uterus
(4) Vascular damage
(a) Related to vaginal and caesarean delivery

**Table 2:** Risk factor for development of VTE.

(1) Previous history of thrombosis
(2) Primary thrombophilia (e.g., factor V Leiden)
(3) Caesarean section delivery esp. emergency section during labour
(4) Sickle cell disease
(5) Mechanical heart valve
(6) Smoking
(7) SLE
(8) Atrial fibrillation
(9) Inflammatory bowel disease
(10) Nephrotic syndrome
(11) Antiphospholipid syndrome
(12) Prolonged immobilization (e.g., bed rest)
(13) Recent major surgery or trauma
(14) Age over 35 years
(15) Obesity (BMI > 30 kg/m^2^)
(16) Multiparity over 4 deliveries
(17) Preeclampsia
(18) Current infection
(19) Complications of pregnancy such as antepartum or postpartum hemorrhage, hyperemesis gravidarum, condition requiring blood transfusion, and fluid-electrolyte imbalance

**Table 3:** Plasma D-dimer levels during pregnancy.

D-dimer (plasma)				
Units	Adult	First trimester	Second trimester	Third trimester
*μ*g/mL	<0.5	0.05–0.95	0.32–1.29	0.13–1.7

*μ*g/L ng/mL	<500	50–950	320–1290	130–1700

nmol/L	<2.7	0.3–5.2	1.8–7.1	0.7–9.3

**Table 4:** Indications for thromboprophylaxis during pregnancy.

(1) Mechanical heart valve
(2) Rheumatic heart disease
(3) Atrial fibrillation
(4) Antithrombin III deficiency
(5) Antiphospholipid syndrome
(6) Prior anticoagulation therapy
(7) Factor V Leiden defect
(8) Prothrombin G20210A mutation

**Table 5:** Unique aspects that need to be considered for thromboprophylaxis during pregnancy.

(1) Transplacental transfer
(2) Expanded blood volume up to 50%
(3) Increase in volume of distribution
(4) Increase in GFR leading to enhanced excretion of heparin
(5) Enhanced protein binding of heparin
(6) Shorter half lives of UFH and LMWH—higher and frequent dose requirement
(7) Risk of heparin-induced thrombocytopenia (also known as HIT)

**Table 6:** Thromboprophylaxis using UFH—dosage and monitoring.

(1) Low-dose prophylaxis
(a) First trimester: 5000 to 7000 Units q12 hours
(b) Second trimester: 7500 to 10,000 Units q12 hours
(c) Third trimester: 10,000 Units q12 hours
(i) Unless aPTT elevated
(2) Adjusted dose prophylaxis to aPTT of 1.5 to 2.5
(a) Dose: 10,000 q8–12 hours
(b) Goal aPTT: 1.5 to 2.5 times normal

**Table 7:** Dose of LMWH as per body weight.

LMWH type	Body weight < 50 Kg	50–69 Kg	70–90 kg	>90 kg
Enoxaparin	20 mg daily	60 mg daily	40 mg twice daily	40 mg twice daily
Dalteparin	5000 U daily	6000 U daily	8000 U daily	10,000 U daily
Tinzaparin	175 U/kg once daily	175 U/kg once daily	175 U/kg once daily	175 U/kg once daily
Rivaroxaban (oral)	Contra indicated			
